# NanoCAGE-XL and CapFilter: an approach to genome wide identification of high confidence transcription start sites

**DOI:** 10.1186/s12864-015-1670-6

**Published:** 2015-08-13

**Authors:** Jason S. Cumbie, Maria G. Ivanchenko, Molly Megraw

**Affiliations:** Department of Botany and Plant Pathology, Oregon State University, Corvallis, OR 97331 USA; Department of Electrical Engineering and Computer Science, Oregon State University, Corvallis, OR 97331 USA; Center for Genome Research and Biocomputing, Oregon State University, Corvallis, OR 97331 USA

**Keywords:** Transcription start site (TSS), Promoter, nanoCAGE, Capped analysis of gene expression

## Abstract

**Background:**

Identifying the transcription start sites (TSS) of genes is essential for characterizing promoter regions. Several protocols have been developed to capture the 5′ end of transcripts via Cap Analysis of Gene Expression (CAGE) or linker-ligation strategies such as Paired-End Analysis of Transcription Start Sites (PEAT), but often require large amounts of tissue. More recently, nanoCAGE was developed for sequencing on the Illumina GAIIx to overcome these difficulties.

**Results:**

Here we present the first publicly available adaptation of nanoCAGE for sequencing on recent ultra-high throughput platforms such as Illumina HiSeq-2000, and CapFilter, a computational pipeline that greatly increases confidence in TSS identification. We report excellent gene coverage, reproducibility, and precision in transcription start site discovery for samples from *Arabidopsis thaliana* roots.

**Conclusion:**

nanoCAGE-XL together with CapFilter allows for genome wide identification of high confidence transcription start sites in large eukaryotic genomes.

**Electronic supplementary material:**

The online version of this article (doi:10.1186/s12864-015-1670-6) contains supplementary material, which is available to authorized users.

## Background

Accurate identification of transcription start sites (TSS) for RNA polymerase II (pol-II) genes is critical for determining promoter location, which in turn facilitates accurate determination of functional sequence control elements [1–3]. While standard RNA-Seq methodologies can provide some insight into the nature of full-length transcripts, a heavy 3′ bias in data outcomes must be addressed with a 5′ cap-trapping strategy. Several overall strategies have been published for application to animal tissues and cell lines; these are well-characterized by three protocols. The Paired-End Analysis of Transcription start sites (PEAT) protocol [4] enriches for capped transcripts in two steps: an initial dephosphorylation of uncapped transcripts, followed by the ligation of a short “tag” sequence to the 5′ ends of capped transcripts. The Cap Analysis of Gene Expression (CAGE) method [5] actively “traps” the 5′ N7-Methylguanosine-triphosphate (^7m^G-p-p-p-N) modification common to all pol-II generated transcripts, known as the “cap”, with streptavidin beads. Both PEAT and CAGE have been widely employed in animal studies [4, 6, 7], and recently we have successfully applied the PEAT strategy to plant tissues [3]. The nanoCAGE protocol aims to reduce the required amount of total RNA from the 50 to 150 μg necessary with PEAT and CAGE to the level of nanograms by using a combination of template switching and semi-suppressive PCR, and has been reported to have a level of sensitivity 1000 times higher than that of CAGE [8, 9].

In contrast to PEAT and CAGE, nanoCAGE does not use restriction enzymes or linker ligation, and is therefore not influenced by restriction enzyme inefficiency, competition for naturally occurring restriction sites, or sequence biases resulting from ligation inefficiency of the 5′ linker. Additionally, nanoCAGE, like PEAT and CAGE, uses random primers as opposed to polyA priming, increasing the chance of 5′ end capture in the case of long transcripts. Challenges remain for broad use in current applications to plant and animal organisms with tens of thousands of genes, as the nanoCAGE protocol was developed for the Illumina GAIIx platform and it is reported to yield approximately one million raw reads per library for multiplexed libraries [8, 9]. NanoCAGE also generates false TSSs due to premature template switching before reaching the very 5′ end of transcripts [9]. While it has not yet been widely applied in published data outcomes, the strategy has demonstrated strong potential for reducing the quantity of input total RNA if sequencing depth and template-switching artifacts can be addressed. Currently, one published study [10] has used the nanoCAGE protocol [8, 9] on the Illumina HiSeq-2000, but little was presented on the methodological differences as the sequencing was done commercially.

In this study using RNA from *Arabidopsis* roots we present the first publicly available adaptation of nanoCAGE for sequencing libraries on the powerful Illumina HiSeq-2000 platform, thus increasing sequencing depth and achieving excellent genome coverage. These samples are ideal for protocol development, as they present a challenging case where both rRNA removal and sequencing depth are critical for appropriate data coverage of a large eukaryotic genome. We develop and analyze a series of important protocol changes, and provide information on barcode effects on library performance. Additionally, we develop an annotation-free computational filter based on identification of TSS peaks (read clusters) derived from capped mRNAs that greatly increases confidence in TSS discovery. Importantly, this computational filter does not require removal of sequenced reads in pre-processing, and therefore allows the end-user to balance sensitivity with precision based on experimental needs. We provide a complete experimental protocol [Additional file 1], an analysis of gene coverage and precision of TSS identification, and description of the computational procedure with software implementation for the identification and removal of false TSSs.

## Results

### Linker sequence and template input in nanoCAGE introduce important trade-offs in sequencing outcome

To assess the viability of the nanoCAGE protocol with different sequencing strategies, we prepared a total of 10 libraries over three separate experiments using both single and barcoded library formats, with or without a linker -- a short six-nucleotide sequence introduced in the template switching (TS) oligo to normalize barcode biases in library preparations [9] -- for sequencing on the HiSeq-2000 platform (Table 1). While the original protocol was developed using total RNA as template [8, 9], we found that using plant total RNA resulted in “spiky” libraries suggestive of very high read redundancy, which was overcome by depleting the rRNA content of our samples [Additional file 2: Figure S1]. The first library sequenced was prepared using the single library format [8], which did not include a barcode or linker sequence in the TS oligo (Table 1). Although replicates are essential for the assessment of method repeatability, our goal with this first experiment was to determine a baseline outcome from the maximal profiling of an individual *Arabidopsis* library. In experiments 2 and 3, we tested barcode combinations in multiplexed libraries including and excluding the linker in the TS oligo respectively (Table 1).

**Table 1:** Summary of sequencing and alignment results

Samples^a^	Adaptor Sequence^b^	Number of Raw Reads (×10^6^)	Uniquely Mapped Reads^c^ (×10^6^)	% rRNA Reads^d^	Redundancy^e^	Average # of Genes with TSS Peaks (×10^3^)^f^	Average # of Peaks per Gene^g^
Exp. 1							
S1BALA	GGG	183	123	0.29	3.73	10	1.23
Exp. 2							
S2BPLP	**ATCGTG** *GCTATA* GGG	61	17	2.90	2.08	15	1.3
S3BPLP	**GATCGA** *GCTATA* GGG	26	5	2.15	1.93	12	1.13
S4BPLP	**TCGAGC** *GCTATA* GGG	37	6	2.44	1.98	13	1.18
Exp. 3							
S5BPLA	**CAGATC** GGG	18	14	16.05	2.84	7	1.13
S6BPLA	**CCGTCC** GGG	15	12	22.80	4.31	10	1.29
S7BPLA	**CGATGT** GGG	17	13	24.91	3.21	8	1.18
S8BPLA	**CTTGTA** GGG	16	12	24.01	4.23	10	1.3
S9BPLA	**GCCAAT** GGG	26	22	27.23	5.58	14	1.5
S10BPLA	**TGACCA** GGG	24	20	23.89	3.76	11	1.29

^a^Samples are formatted with the sample id (S#) followed by either BA (barcode absent) or BP (barcode present), and end with either LA (linker absent) or LP (linker present)

^b^Sequence from the template switching oligo present at the start of reads: barcodes are in bold, linker in italic, the 3G-tail is underlined

^c^Number of reads that map to unique genomic loci

^d^Percent of reads that mapped to ribosomal RNA (rRNA)

^e^Calculated as (number of uniquely mapped reads)/(number of unique read sequences)

^f^Calculated as the number of protein coding genes with at least one transcription start site (TSS) peak (after G’-filtering)

^g^Calculated as the average number of G’-filtered TSSs found per gene for genes in column ‘f’

#### Read mappability

**Fig. 1:**
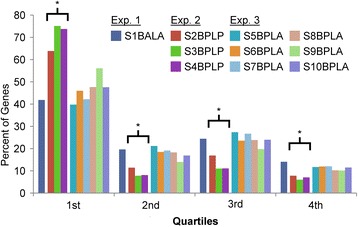
Percent of genes with the majority of reads mapping within each gene quartile. All identified protein coding genes were divided into four even quartiles numbered from 5′ - > 3′ position relative to the gene sequence. The number of reads whose 5′ most base resided in a given quartile (x-axis) was counted, and the percent of genes (y-axis) with the majority of reads in a given quartile was plotted. The total number of genes analyzed was: 25842, 21804, 20552, 20909, 20567, 19095, 20438, 19432, 19733, and 20785 for samples S1BALA, S2BPLP, S3BPLP, S4BPLP, S5BPLA, S6BPLA, S7BPLA, S8BPLA, S9BPLA, and S10BPLA respectively, with 22468 and 22223 genes analyzed for samples in experiment 2 and 3 combined, respectively. Bracketed bars with a ‘*’ symbol denotes libraries prepared with a linker sequence in the template switching oligo

For all experiments, analyses focused on protein coding genes representing ~27,000 genes in *Arabidopsis*. In experiment 1, ~67 % of the raw reads (Table 1) could be mapped to unique loci associated with over 25,000 genes (Fig. 1), providing a large number of reads that could be further used in TSS peak calling. In experiment 2 we used barcodes selected from [9] with the linker present for three separate libraries (Table 1), in order to test the viability of multiplexing data on the Hi-Seq 2000 platform. It was previously reported that the introduction of a low-“G” linker sequence in the TS oligo or the use of an *in silico* post-alignment filter could reduce barcode-specific biases resulting from strand invasion artifacts [9] -- that is, reads produced from premature template switching caused by the TS oligo “invading” the growing cDNA strand due to sequence complementarity. To test whether this was the case in our data, we used a similar *in silico* approach to remove strand invasion artifacts by examining the genomic complementarity of each TS oligo as described in [9]. We found a generally small effect of strand invasion, although libraries without the linker sequence had slightly more than twice as many reads with evidence of strand invasion (~0.9 % of all reads on average for libraries prepared without the linker compared to ~0.4 % of all reads for libraries prepared with the linker). However, the introduction of both a barcode and a linker sequence came at a dramatic cost to read mappability: an approximately three to four-fold reduction in the percent of mappable reads as compared to experiment 1 (Table 1). The presence of a linker sequence that is identical for all reads markedly reduces read diversity resulting in dramatically lower sequencing accuracy, a well-documented problem on Illumina sequencers ([8]; Update to Article]. The number of genes associated with mapped raw reads was also reduced in experiment 2 (20,000-21,000 genes per library) as compared with experiment 1 (~25,000 genes) (Fig. 1). The numbers of genes per library with high-confidence TSS read clusters (peaks) obtained after filtering, however, were very good as compared to experiments 1 and 3 (Table 1); this point is discussed later in results.

In experiment 3, we adapted the Illumina TruSeq barcodes and used them as per Illumina’s TruSeq sample pooling guide [11]. We selected barcodes containing as few “G”s as possible in order to avoid barcode biases caused by strand invasion [9], and pooled six different libraries to increase sequence diversity and address the low mappability observed in experiment 2. The linker was removed in order to evaluate whether using Illumina-recommended barcodes would improve read mappability. As expected, read mappability improved: a slight increase in percent of mapped raw reads was observed as compared to experiment 1, and a four to five-fold increase as compared to experiment 2 (Table 1). Experiment 3 had a comparable number of mapped genes to experiment 2 (Fig. 1).

#### Read distribution

While high read mappability is desirable, a representative genomic read distribution is also essential. If too many reads are generated from strand invasion or other artifacts (i.e., noise), then the number of true TSS reads needed to produce identifiable TSS peaks (read clusters) will not be sufficiently large even with high read mappability. To further explore this relationship, we used a peak calling algorithm developed for TSS-Seq reads [4] to analyze the distribution of reads along the length of annotated genes without filtering any of the peaks called. In experiment 1, we found that because of greater sequencing depth, the number of genes with an identifiable peak within 500 bases of the TAIR10 [12] annotated TSS or in the 5′ untranslated region (5′ UTR) was slightly greater than with libraries that were multiplexed: 15,000 genes for experiment 1, as compared to 13,000 and 11,000 genes on average for experiments 2 and 3 respectively (data not shown). Additionally, in all experiments we found an inconsistent average number of peaks per gene prior to peak filtering: 1.9 for experiment 1, 1.2-1.5 for experiment 2, and 1.5-1.7 for experiment 3. To assess the source of this variation in average peak number, we plotted the read distribution along the normalized length of a gene within each library (Fig. 1). We found that those libraries which incorporate a linker sequence (experiment 2) and utilize more input template in their preparation display a much greater bias for reads towards the 5′ ends of genes. At the same time, while these libraries with more template have a 5′ read bias, they also have fewer peak calls at the 5′ ends of genes. This is one indicator that fewer false positives are identified in these libraries, which is confirmed in further analyses discussed in the “CapFilter” section below. Overall, our analysis illustrates that inclusion of the low-“G” linker sequence and use of more template together served to reduce the number of reads resulting from strand invasion and other artifacts.

#### Genome coverage

As expected, the overall sequencing depth in experiment 1, which sequenced one library in one lane, was greater than in experiments 2 and 3 based on the total number of genes with mappable reads (Fig. 1). However, when peaks were filtered in order to identify the most confident TSS peaks, we found that libraries in experiment 2, which utilized the low-“G” linker plus the optimal template input, performed best. On average, we identified 10,000 - 11,000 genes with a high confidence TSS peak per library in experiments 1 and 3, as compared to 13,000 genes in experiment 2 (Table 1). Experiment 3 libraries, which were prepared with less mRNA input and had higher rRNA content in the template due to the presence of total RNA, also had higher rRNA contamination in the library (16 % to 27 %), apparently contributing to a reduction of the number of productive reads and an increase in read redundancy. This is in contrast to much lower contamination level overall for experiments 1 and 2 (0.29 % and 1.93 - 2.08 % respectively) (Table 1).

**Fig. 2:**
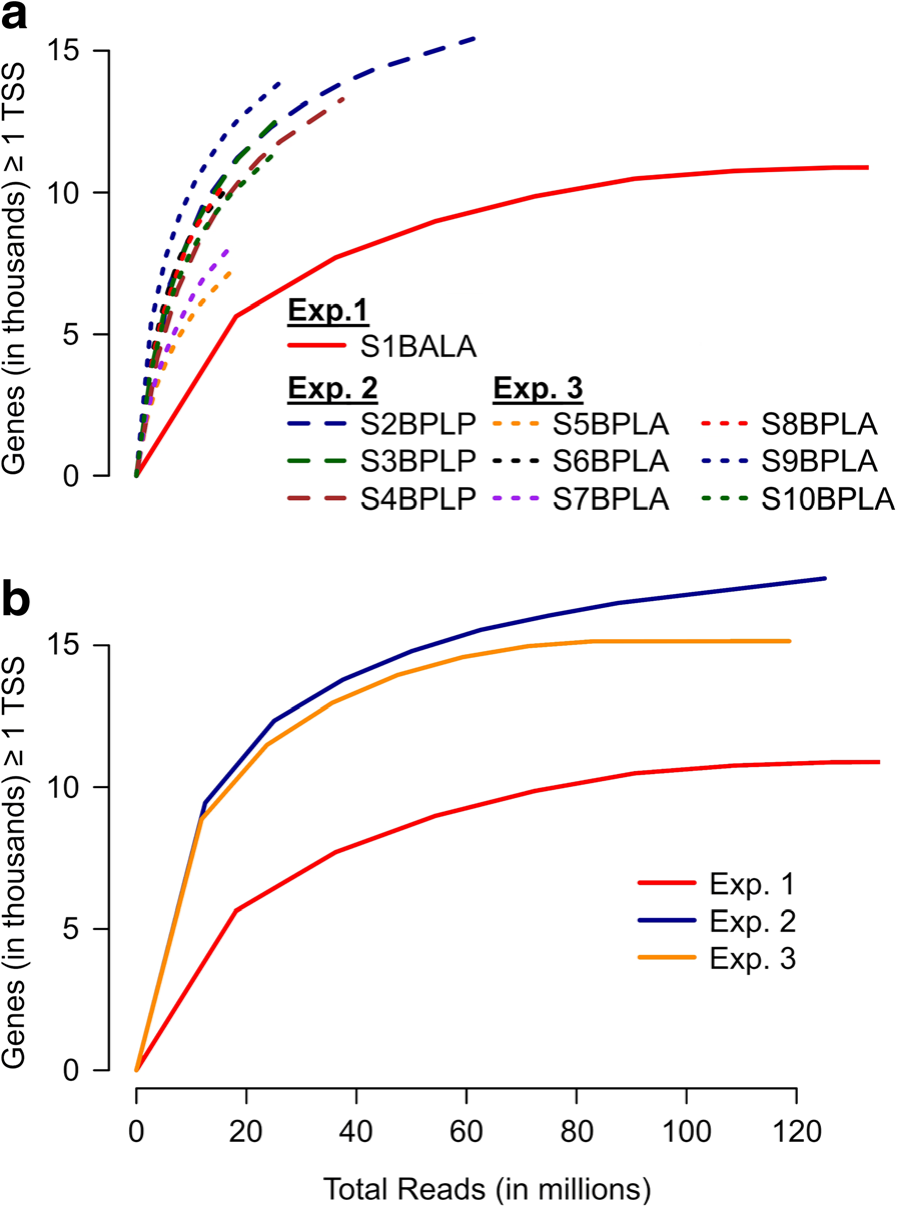
Total genes with at least one high-confidence transcription start site (TSS) after random subsampling. For each library individually (Panel **a**) or for all libraries within each experiment combined (Panel **b**), from 10 to 70 % of reads were randomly subsampled in increments of 10 %. The number of reads (in millions) subsampled for each library/experiment was plotted (x-axis). The reads in each subsample were then mapped to the genome, and the total number of genes with at least one high-confidence TSS peak was computed (y-axis)

To fully illustrate the sequencing depth achieved with the nanoCAGE-XL protocol, and the differences between the three experiments, we randomly subsampled, aligned, and called peaks for the reads produced by each library individually (Fig. 2a) as well as for all reads within each experiment combined (Fig. 2b). For individual libraries, we observed a clear correlation between each barcode’s performance and its “G” content and/or “G” proximity to the 3G-tail of the TS oligo (Table 1), with library S9BPLA performing best and libraries S5-7BPLA and S1BALA performing worst (Fig. 2a). In combined libraries, experiment 2 (less mRNA input) began to level off in the number of new genes identified with a high-confidence peak at 60 million reads, whereas experiments 1 and 3 leveled off closer to 80–90 million reads. The number of genes associated with a high confidence peak in combined data within experiments that were multiplexed (experiments 2 and 3) was slightly higher than for individual libraries (Table 1) within the same experiment (experiment 2: 11,000 to 14,000 individually and 16,000 combined, experiment 3: from 7000 to 13,000 individually and 15,000 combined). This last observation is especially important, since combined datasets began to reach saturation at 15,000 to 16,000 genes, which is at least 2000 to 3000 more genes than were covered using PEAT; 5000 to 6000 more genes were covered than using PEAT when only considering high confidence PEAT peaks filtered based on the criteria used in [3]. Clearly this coverage could not be achieved on the older GAII platform, which generates on average only ~20 million reads per lane. Moreover, the sampling depth showed no barcode bias for experiment 2, which was performed with the linker added; this is in contrast to the significant differences between sampling depth biases for different barcodes in experiment 3, in agreement with previous findings [9]. Overall we find that libraries prepared without the low-“G” linker sequence provided greater read mappability and gene coverage genome-wide. However, this came at the cost of a suboptimal read distribution along the length of the gene for some of the barcodes, e.g., in libraries S5BPLA and S7BPLA, generating fewer genes with a high confidence TSS peak (read cluster) (Table 1).

### Cap signature identifies high confidence TSS peaks

**Fig. 3:**
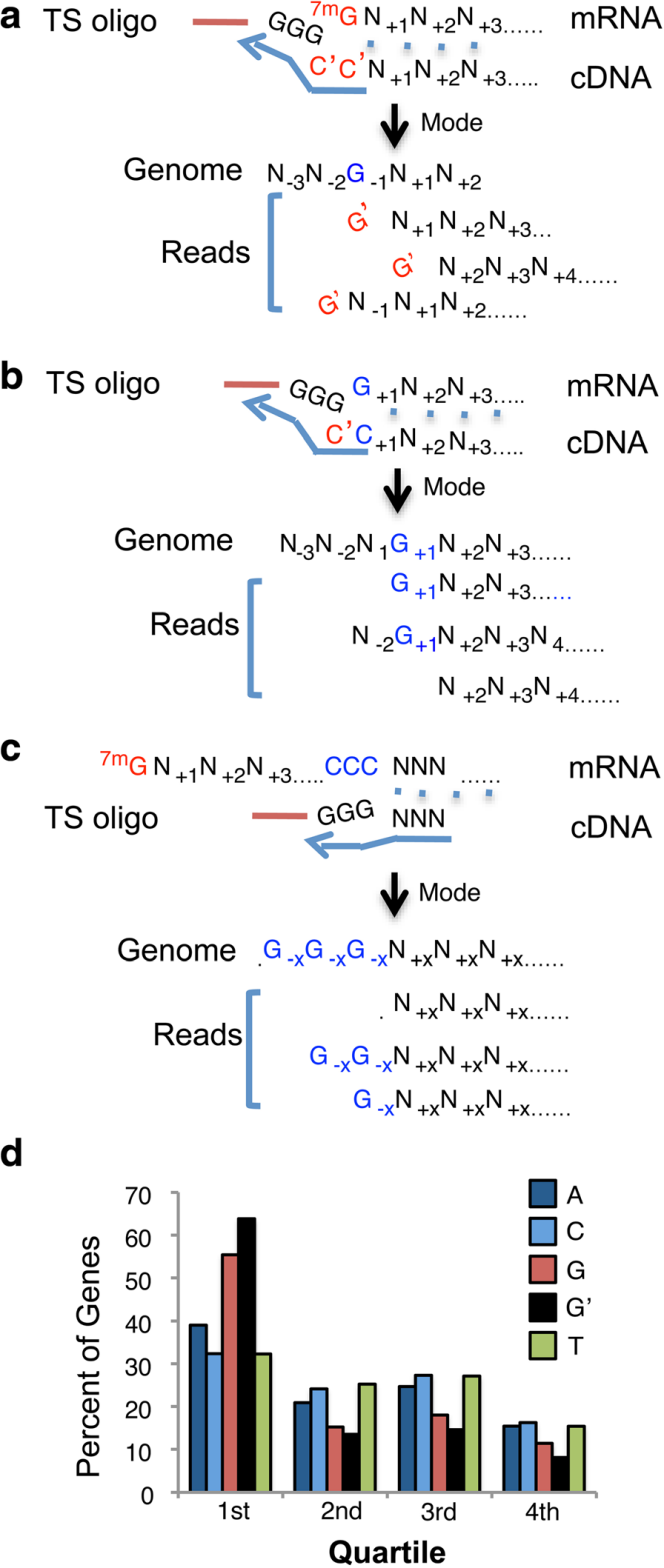
Template switching (TS) overview and layout of read distribution based on read start base. Panel **a:** Canonical expectation – the reverse transcriptase (RT) transcribes the cap structure as an unencoded cytosine, adds one to two additional cytosines beyond the cap structure, and switches templates. When the 3”G” tail of the TS oligo anneals to these additional cytosines, many of the resulting reads will start with a “G” that does not map to the genome. Some of the unencoded “G”s will artificially appear as encoded if they align to genome-encoded “G”s. Letters colored in red represent bases not found in the genome, letters in blue or black represent encoded nucleotides. An arrow indicates the position of the peak mode, i.e., the most-frequently sequenced starting nucleotide. Panel **b**: Non-canonical expectation – template switching in which an uncapped RNA starting with a regular “G” is used. Panel **c**: Diagram of strand invasion that occurs as a result of RNA complementarity to the TS oligo. Panel **d**: All identified genes were divided into four even quartiles numbered from 5′ - > 3′ position relative to the gene sequence. The number of reads whose 5′-most base resided in a given quartile was counted, and the percent of genes with the majority of reads aligning to that gene quartile was plotted. As expected, more reads mapped toward the 5′ end of genes, particularly reads that began with a “G” either encoded (G), or not encoded in the genome (G’)

Because of the ability of the reverse transcriptase (RT) enzyme to transcribe the cap structure [13], many sequenced reads which are derived from capped mRNAs will begin with an unencoded “G”, a “cap signature” which has been previously reported in both CAGE and nanoCAGE data sets [9, 14, 15]. In nanoCAGE, unencoded “G”s will be incorporated when the TS oligo anneals to cytosines beyond the cap-derived cytosine that have been added by the RT’s template free activity (Fig. 3a). Assuming an equal distribution of all four bases in the genome sequence immediately upstream of the start of such reads, only ~25 % of the cap-derived “G”s will align to “G”s encoded in the genome. These artifactually encoded “G”s will be indistinguishable from all other reads starting with an encoded “G” (Fig. 3a). In comparison, false TSS peaks resulting from uncapped or truncated mRNAs starting with a genome-encoded “G” (a non-cap “G”), may also show a bias for reads starting with a “G”-- but most of these non-cap 5′ “G”s will match the reference genome (Fig. 3b, c).

Because of this cap signature, namely the presence of unencoded 5′ “G”s, many reads derived from capped mRNA may fail to align to the genome, potentially resulting in a loss of the most informative reads. In agreement with this expectation, for all reads that began with a “G”, in ~37 % of these cases the “G” was unencoded. This is in contrast to reads beginning with any other base where collectively an average of ~4 % of those cases began with unencoded instance of that base [Additional file 2: Figure S1 and Additional file 3: Table S1]. This average dropped to ~2 % for all libraries at the second nucleotide, regardless of base [Additional file 2: Figure S1 and Additional file 3: Table S1]. This indicated that a “C” complementary to the 5′ cap was frequently incorporated and converted into a “G” during the second strand synthesis in reads derived from capped mRNA, with additional nucleotides much less likely to be incorporated. The percentage of unencoded “G”s was on average higher for the libraries in experiment 2 (~66 %) as compared to experiments 1 and 3 combined (~24 %) [Additional file 2: Figure S1 and Additional file 3: Table S1], suggesting that using the low-“G” linker in addition to having a more optimal mRNA template input increases the chances of the TS-oligo to anneal to the cap-derived cytosine (see Fig. 1). To overcome the potentially lower mappability of reads derived from capped mRNAs, we removed the first base from each read prior to alignment, while keeping track of the removed base. When examining read alignments relative to their position along the length of genes, a bias was observed toward reads mapping to the 5′-most portion of the gene regardless of their beginning base (Fig. 3d). This was likely due to the TS oligo annealing directly to the cap-derived cytosine (Fig. 3a). However, the bias for reads starting with a “G”, either encoded or unencoded, was remarkably pronounced within the 5′-most portion of the gene. Thus, reads beginning with this “cap signature” display this strong “G”-bias, and are more likely to be true TSSs. This observation allows for the development of filtering approaches for the elimination of potential false positives and other background noise in downstream analyses.

### CapFilter: a software program to provide G’-filtering of peaks allows for annotation-free TSS identification

**Fig. 4:**
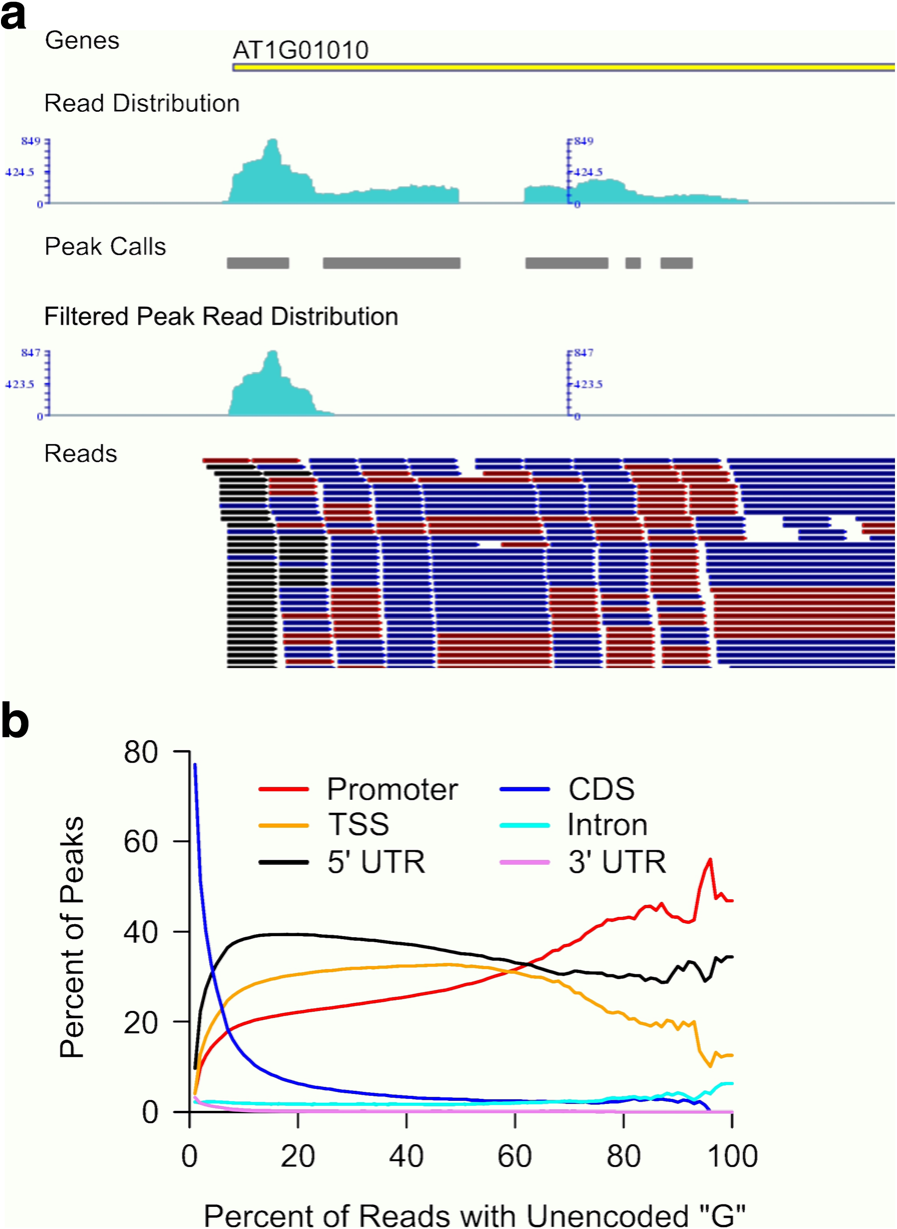
Illustration of the procedure of G’-filtering. Panel **a**: A GBrowse [39] snapshot of a representative location showing filtered and unfiltered TSS-Seq data. The track labeled “Reads” displays individual reads mapped to the genome, with those beginning with a “G” in red, those that begin with a “G” that does not match the reference genome (unencoded) in black, and all other reads in blue. The track labeled “Peak Calls” shows all peaks called using all reads from the “Reads” track. The track labeled “Read Distribution” shows a histogram of all reads found in the “Reads” track, while the “Filtered Peak Read Distribution” track shows a histogram of the reads belonging to peaks that passed G’-filtering. The “Genes” track shows genome annotation for the gene (AT1G01010). Panel **b**: Representative distribution of TSS peak calls along the length of genes based on percent of reads starting with unencoded “G”s. The percent of reads in each peak that began with a “G” that did not match the reference genome was calculated and peaks were filtered based on having a minimum of 0 to 100 % of reads beginning with an unencoded “G” (x-axis). All peaks passing the G’ filter were then categorized based on the gene part to which they aligned: promoter = ≤3000 bp upstream from TAIR10 annotated TSS, TSS = peak overlaps TAIR10 TSS, 5′ UTR = peak begins in the 5′ untranslated region, CDS = peak begins in the coding portion of a gene, Intron = peak begins in an intron of the gene, 3′ UTR = peak begins in the 3′ untranslated region. The percent of peaks annotated for each category with the minimum percent of reads beginning with an unencoded “G” was then totaled and plotted (y-axis)

**Fig. 5:**
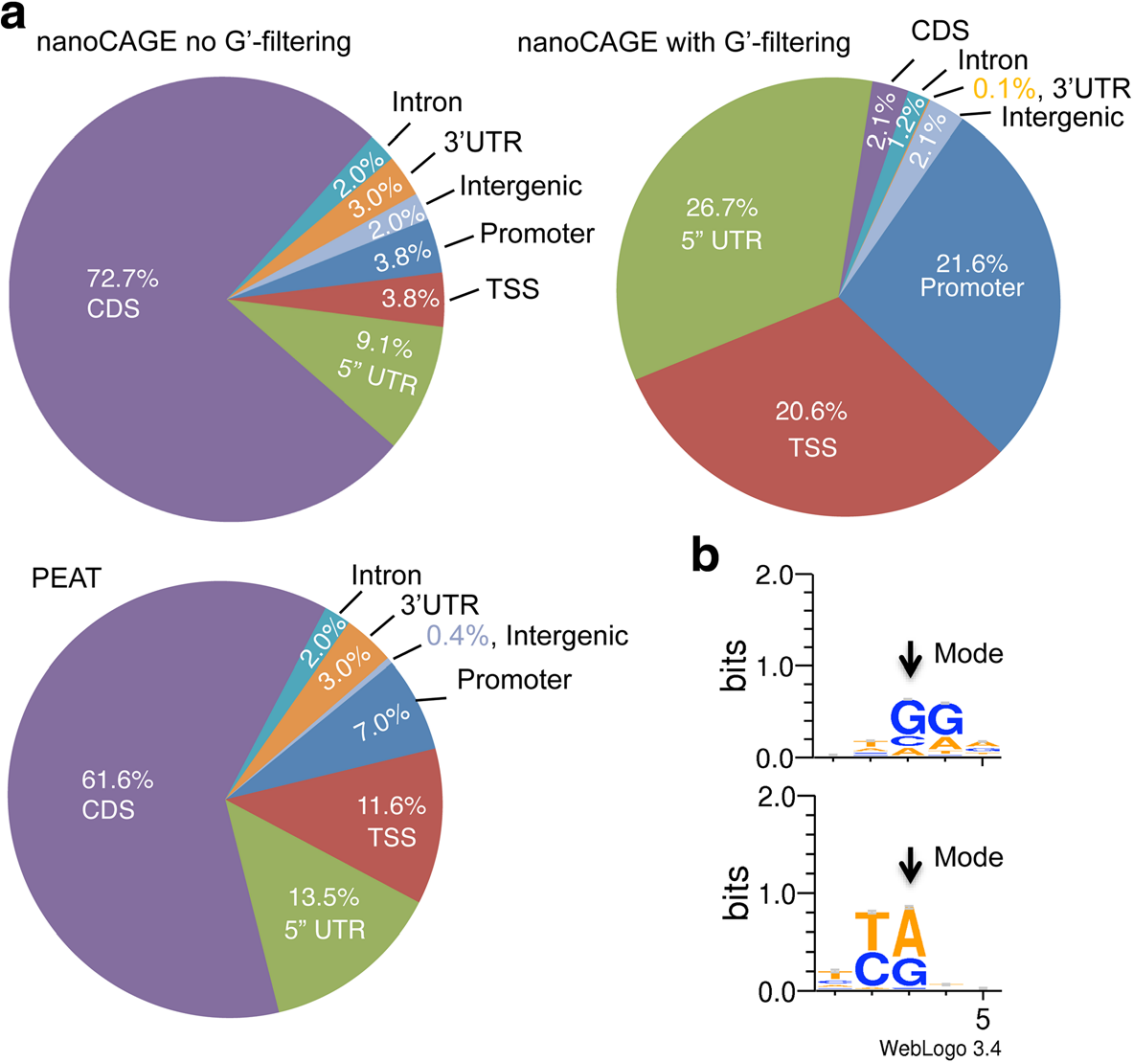
Peak call distributions and word logos at peak modes showing the effect of G’-filtering. Panel **a**: Distribution of peak calls for nanoCAGE before (left) and after G’-filtering (right), and for unfiltered PEAT peak calls (bottom). Presented are results for library S1BALA (experiment 1). Promoter = ≤3000 bp from TAIR10 transcription start site (TSS), TSS = overlapping TAIR10 TSS, 5’ UTR = 5′ untranslated region, CDS = Exons minus untranslated regions, Intron = intronic regions, 3′ UTR = 3′ untranslated region, Intergenic = peaks > 3000 bp from a TSS or > 1000 bp from 3′ portion of gene. Panel **b**: WebLogo 3 [18] was used to produce word logos at the peak modes for all peaks (top) or only those peaks that passed filtering (bottom). An arrow indicates the position of the read peak mode

An important feature of TSS-Seq reads is that they do not originate from one or even a few nucleotides, but “cluster” together in distinct peaks or “initiation patterns”, which can then be used to identify the most strongly preferred TSS locations for a gene [3, 9, 16]. Using a peak calling procedure developed in [4], we identified peaks in regions near gene start sites as annotated in TAIR10 [12]. However, many reads in our data also formed peak-like clusters within other annotated portions of the gene, indicating that some peaks could be false positives generated from artifacts (Fig. 4a). The *in silico* filtering mechanism provided in [9], intended to eliminate strand invasion artifacts arising from genomic complementarity of the TS oligo used in library construction, still left the distribution of peaks called along the length of the gene virtually unchanged in our data. This was true even when relaxing the parameters of the procedure to allow for more than 5 mismatches (data not shown). To achieve a reliable selection of TSS peaks derived from capped mRNA, we developed a new filtering mechanism implemented in our CapFilter software that identifies and filters peaks based on the “cap signature”, which we refer to as “G’-filtering”. The procedure for G’-filtering is straightforward: it calculates the percent of reads within a peak that begin with an unencoded “G”, and selects the high-confidence TSS peaks based on this percentage. We found that as we increased the minimum percent of unencoded “G”s required to pass the G’-filter beyond 15 %, the overall distribution of peaks trended markedly toward annotated locations corresponding to the 5′ portions of genes (Fig. 4a, b). Since the peak calling program [4] used by CapFilter takes a cut-off at 10 reads per peak, all identified peaks contain a sufficient number of reads for applying G’-filtering. When we tested a stringent cutoff of 50 %, those peaks most likely to be spurious (i.e., within the coding region, an intron, or 3′ UTR) dropped from 70 to 80 % of all peaks to no more than 5-10 % for all samples in all three experiments, indicating that this cutoff would provide a simple and reliable filter for identifying confident TSS candidates derived from capped mRNA (Fig. 5a; Additional file 2: Figure S2). The estimated false negative rate, i.e., the percent of rejected TSSs at true 5′ locations, ranged from 7 to 11 % across all samples (Additional file 4: Table S2). Furthermore, we applied G’-filtering to an unrelated set of mammalian raw data obtained via the classical cap-trapping CAGE protocol available in the literature [17], and also observed improvement in the TSS peak locations (Additional file 2: Figure S3).

As an additional measure for testing the precision of our G’-filtering approach, we generated sequence logos using Web Logo 3 [18] from the nucleotides surrounding the peak mode, the genome coordinate most commonly sequenced in a peak, to identify enriched nucleotides at the most-preferred TSS positions. Previous research has shown that in plants there is a preference for a pyrimidine (Y: C or T) at the −1 nucleotide, i.e., the nucleotide immediately upstream of the TSS, and a purine (R: A or G) at the +1 position, which is referred to as the “YR rule” [19]. We found that prior to G’-filtering, the +1 and +2 nucleotides were enriched primarily for a “GG” motif (Fig. 5b). This occurred even when using the *in silico* filter for strand invasion artifacts developed in [9], indicating that the presence of “C”-rich regions alone in mRNA without additional complementarity to the TS oligo may be sufficient for promoting strand invasion. After G’-filtering, the primary bases enriched at the −1 and +1 nucleotide positions were “T,C” and “A,G”, respectively, consistent with the “YR rule” [19], indicating that G’-filtering was indeed capturing peak modes that were high confidence TSSs in our data set. In summary we find that the use of CapFilter greatly enriches for peaks composed predominantly of true TSSs. These CapFilter results include a non-negligible portion of low-coverage peaks (10–20 reads), highlighting its ability to capture true TSS peaks regardless of coverage.

### NanoCAGE-XL is highly reproducible after G’-filtering

Reproducibility is an important metric for the quality of TSS-seq. To address the reproducibility of our nanoCAGE-XL data, we examined the correlation in read coverage for individual peaks, the consistency between TSS peak modes (the position most commonly sequenced in a read cluster) of biological replicates, and the consistency between nanoCAGE-XL peak modes and those in a PEAT data set generated using the same sample type [3].

#### Correlation analysis

For the read coverage correlation analysis, we used a similar approach to that in [9] to perform pair-wise comparisons for all of our data sets [Additional file 2: Figure S1 and Additional file 5: Table S3]. Briefly, the number of reads per million (RPM) was calculated for each peak in one library of our data set, and this number was compared to the RPM of the corresponding peak in a separate library using a Spearman’s rank correlation analysis. Peaks were considered to be corresponding based on the overlap of their start/end genomic coordinates. The average Spearman’s rho of 0.67 showed a moderate level of positive correlation, a value in agreement with previous levels of correlation reported for nanoCAGE [9]. This value was even greater for comparisons between libraries using the common linker sequence (average Spearman’s rho of 0.88) [Additional file 2: Figure S1 and Additional file 5: Table S3], indicating that the common linker reduced biases based on barcode difference, consistent with results reported in [9].

#### Peak mode analysis

**Fig. 6:**
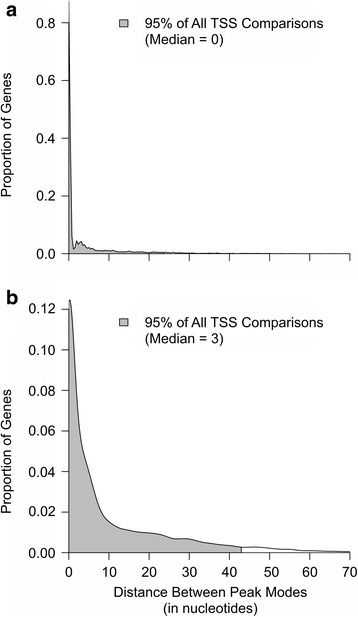
Representative sample of distribution of peak mode differences. Peaks were identified and filtered for all reads in experiment 2 and experiment 3 of the nanoCAGE samples (Panel **a**) or experiment 2 and PEAT (Panel **b**). Similar results were found for all other comparisons. The peak modes were compared for peaks whose start and end coordinates overlapped. A kernel density plot is used to depict the proportion of genes (y-axis) whose peak modes were within “X” nucleotides of each other (x-axis). The shaded area under the plot represents 95 % of all peak modes that were compared

To evaluate how consistently TSS peak modes were identified across nanoCAGE-XL libraries, and how they compared to PEAT data obtained in [3], we first identified the corresponding peaks between data sets. We then calculated the median and average distances between corresponding peak modes for all pair-wise comparisons. We include both the median and average calculations to more accurately describe the distribution of peak mode differences in each pair-wise comparison. While the average distance uses a summation of all values for its calculation, it is more strongly affected by outliers than the median. In contrast, the median provides a single data point to represent the maximum and minimum value of the lower and upper 50 % of the data respectively. For any given pair-wise comparison, this accounted for 8000 to 14,000 genes with peaks prior to G’-filtering or 4000 to 12,000 genes with peaks post G’-filtering (data not shown), indicating that most genes had at least one peak that overlapped in each pair-wise comparison both before and after filtering. The median distance calculated was between 0 and 2 nucleotides for all pair-wise comparisons within nanoCAGE-XL data sets; this median distance dropped to zero when employing G’-filtering, indicating that for most peaks the same peak mode was called. The average distance between peak modes ranged from 5 to 18 nucleotides, which dropped to 3 to 7 nucleotides after applying G’-filtering; at least 95 % of peak modes found were within ~30 nucleotides of each other in our nanoCAGE-XL data (Fig. 6a). When comparing nanoCAGE-XL to PEAT, we found that the median distance between peak modes ranged from 3 to 6 nucleotides, with 2 to 5 nucleotides after applying G’-filtering (Fig. 6b). The average distance between peak modes ranged from 12 to 17 nucleotides, with 10 to 12 nucleotides after G’-filtering. For PEAT data comparisons, we used only those peaks located within 500 bp of the TSS or within the 5′ UTR as in [3], since the PEAT protocol removes the 5′ cap prior to library construction preventing G’-filtering of the obtained peaks. Thus nanoCAGE-XL shows remarkably high reproducibility at single base resolution across biological replicates, and produces TSS peaks and peak modes comparable to those of PEAT despite a very different technical approach.

## Discussion

Correct identification of where transcription begins is a critical factor in studying both the process of transcription itself as well as its regulation. For example, due to the degenerate nature of transcription factor binding sites, identifying their precise genomic locations is especially difficult without first correctly identifying their corresponding TSSs [2, 3]. Our previous work has demonstrated the feasibility of this task using bioinformatically selected PEAT data sets combined with logistic regression models that incorporate spatial sequence information for well characterized transcription factors and their respective binding sites [2, 3]. However, the use of PEAT TSS-Seq data precludes the analysis of samples where limited tissue is available due to the high RNA input requirement for this protocol. Additionally, the PEAT protocol removes the 5′ cap before library construction, which prevents noise filtering based on the presence or absence of a cap signature. NanoCAGE dramatically reduces the amount of input RNA needed to interrogate transcription start sites genome wide, although this comes at the expense of low sequencing depth and genome coverage [8, 9]. We were able to address these caveats in the nanoCAGE-XL protocol, and to show that nanoCAGE-XL is appropriate even for plant samples, a notorious technical challenge due to high rRNA content. All raw sequencing data and TSS peak calls are publicly available in the Short Read Archive (SRA) [20]. By upscaling library preparation, using rRNA-depleted templates, and selecting appropriate barcodes, we were able to prepare libraries suitable for sequencing on Illumina’s HiSeq-2000 platform while achieving excellent genome coverage in *Arabidopsis* root samples. Commensurate with RNA-Seq studies [21] and microarray studies [22] in developing whole *Arabidopsis* root samples, as summarized in [3], our nanoCAGE-XL libraries covered ~20,000 of the ~27,000 total protein coding genes in the *Arabidopsis* genome; over 16,000 of these genes have a high confidence TSS peak. Importantly, using the cap signature common in nanoCAGE reads that are derived from capped mRNAs allowed us to develop a new bioinformatic filtering methodology for the identification of true TSSs.

We found that considerations related to library barcoding must be taken into account when preparing nanoCAGE libraries for HiSeq-2000. In the non-barcoded libraries, the presence of a 3G-tail at the start of each read generates low read diversity that is detrimental for HiSeq-2000 sequencing, as compared to sequencing on the older GAIIx platform originally used by nanoCAGE [8]. Introducing inline barcodes, increasing the number of differentially barcoded libraries per lane, and using Illumina-recommended barcode combinations overcomes this problem and increases the number of mappable reads generated. A potential further improvement is to use indexing that incorporates the barcode sequences at the end of reads and requires a separate sequencing step, which has recently been introduced in a number of Hi-Seq 2000 applications. However, such an approach would also require the design of a custom sequencing primer that would anneal over the 3G-tail of the TS oligo. Generally, primers with high G/C content at their 3′ end do not perform well in PCR-based applications. Therefore, such an approach has not yet been tested.

During the RT step employed in generating first strand cDNA in nanoCAGE, two events occur: 1) the RT enzyme incorporates a cytosine nucleotide complementary to the 5′ cap of capped mRNA [13], and 2) one to two additional cytosine bases are commonly added beyond the 5′ cap by the enzyme’s template-free activity [13, 23–25]. In both nanoCAGE and CAGE, the unencoded “C”s are converted into “G”s in the final sequenced reads, creating a “cap signature” [9, 14–16]. This is in contrast to the PEAT protocol, which removes the cap prior to library construction [3, 4]. In agreement, we found that nearly 40 % of our raw reads started with a “G” that did not match the reference genome. While a small portion of this unencoded “G” bias may arise from sequencing error, the fact that the remaining portion of the same reads matched the reference genome suggests that the majority of these “G”s represent the “cap signature”. We took advantage of this easily identifiable marker that distinguishes reads generated from capped mRNA products to develop a computational approach that selects for true TSS peaks. While it may be intuitive to simply select for reads starting with an unencoded “G” and disregard all other reads, we found that a large number of peaks located near the 5′ ends of TAIR-annotated genes also had a high proportion of reads that began with encoded “G”s. For these encoded “G”s it is impossible to determine whether they are cap-derived or instead arise from artifacts such as strand invasion. Therefore, instead of selecting for reads beginning with unencoded “G”s, we based our filtering approach on selecting for TSS peaks that are marked with a high proportion of reads starting with unencoded “G”s.

We also investigated a threshold that could be used to efficiently filter for high-confidence TSSs by examining the proportion of reads with unencoded “G”s which mapped within annotated genes. We determined that a cutoff of 50 % of such reads within a peak was stringent enough to identify high confidence TSS peaks and was sufficient to eliminate artifactual peaks. Using this cap-signature filter is especially effective because 1) it does not require *a priori* genome annotation, and 2) it applies to all peaks, regardless of coverage or position along a gene’s sequence. This second point is especially important since some of the TSS peaks identified within our data sets after G’-filtering, as well as those reported for PEAT samples [3] or other organisms as diverse as human [14, 16], Drosophila [26] and zebrafish [7], map to unexpected gene regions (e.g., the 3′ UTR or the coding sequence) or outside of genic regions entirely. In all of these cases, the presence of novel or unexpected transcription starting sites is an open question. The simple procedure of G’-filtering that we report here should provide an unbiased approach for examination and filtering of genuine TSSs in many organisms as long as the experimental protocol preserves the cap signature, as in nanoCAGE [8] and CAGE [16]. The stringency of this filter can be modified depending on experimental needs. For example, stringency can be lowered to allow for increased gene coverage or higher representation of non-canonical TSS locations, bearing in mind the trade-offs with increased false positive risk.

## Conclusions

In this paper we introduced the first publicly available protocol adapting nanoCAGE for the HiSeq-2000 sequencing platform, making TSS sequencing of low input samples practical where significant depth of coverage is required. Using CapFilter, we were able to demonstrate that the reproducibility of nanoCAGE-XL TSS peak calls was very high, with identical peak mode positions found for a substantial portion of all peaks. All cases of non-identical peak mode positions fell within a short distance of each other, indicating that nanoCAGE-XL with CapFilter achieves truly nucleotide level resolution for identified TSS peaks genome wide. Not only is this method precise and internally reproducible, but peak comparisons show a robust cross-platform reproducibility with the PEAT protocol for peaks with moderate to high coverage, although PEAT does not allow for G’-filtering. For loci with low coverage, our analysis suggests that CapFilter provides an advantage in detecting peaks predominantly composed of capped transcripts. When combining nanoCAGE-XL libraries within a given experiment, we find that nanoCAGE-XL reaches saturation at a higher number of genes than PEAT, highlighting the possibility for greater coverage with nanoCAGE-XL for low input samples.

## Methods

### Plant material and growth conditions

Roots of 9 day-old Col-0 plants grown on vertically oriented agar plates were used in all experiments. Seeds were surface-sterilized in 20 % commercial bleach for 30 min, and rinsed four times for 10 min with sterile water. Sterilized seeds were planted on media containing 1X Murashige and Skoog basal medium with vitamins [27], 1 % sucrose, 10 mM MES buffer pH 5.7 and 0.8 % agar. Plates were incubated at 4 °C for 2 days to ensure even germination after which plants were grown at 21 °C under continuous light conditions with light intensity of 26 μE m^−2^ s^−1^.

### Library construction, quantification and sequencing

Tissue was ground in liquid N_2_ and RNA extracted with TRIzol reagent [28]. Total RNA (RIN 9.0 to 9.6) was treated with 1X RNA Secure Reagent [29] at 65 °C for 10 min, treated with DNase I [29] for 10 min at 37 °C, and purified with RNAeasy kit [30] following the manufacturer’s instructions. rRNA was depleted with Ribo-Zero Magnetic kit (Plant Seed/Root) [31] following the manufacturer’s instructions. Purity and concentration of the resulting mRNAs was determined using Bioanalyzer [32].

RT was performed as in [8] except that either 40 μl reactions with ~200 ng mRNA (experiments 1 and 3), or 20 μl reactions with 50 ng mRNA + 100 ng total RNA (experiment 2) were performed. Reactions were purified with Agencourt RNAClean XP kit [33] as per the manufacturer’s instructions, and first strand cDNA eluted from the beads with 80 μl H_2_O. The optimal number of semisuppressive PCR cycles, usually between 17 and 25, required for second-strand synthesis were determined by quantitative real-time PCR as in [8]. No-template controls were included at this step to test for potential contaminants. Next, for each library, 400 μl semisuppressive PCR reactions were performed using 60 μl of first strand cDNA and the selected number of cycles, after which the reactions were purified with Agencourt AMPure XP kit [33]. Concentration of PCR products was determined with Qubit dsDNA HS Assay kit [34], and products diluted to 10 ng/μl. For each library, addition of sequencing adaptors was performed in 700 μl PCR mixtures. At this step, the ExTaq polymerase was replaced with Phusion Hot Start II High-Fidelity DNA Polymerase [35] in order to generate blunt cDNA ends. The cycling conditions were: 1) 98 °C for 1 min, 2) 1 cycle of 98 °C for 15 s, 55 °C for 10 s, 68 °C for 2 min, 3) 9 cycles of 98 °C for 15 s, 65 °C for 10 s, 68 °C for 2 min. After completion of the PCR, remaining primers were purified by Exonuclease I digestion as in [5]. Namely, 5 μl of Exo I (20 U/ μl) [36] was added per 700-μl PCR mixture and the mixtures incubated at 37 °C for 30 min. Each 700 μl PCR mixture was then mixed with 3.5 ml (5 V) of PB buffer, purified by running through a single column of QIAquick PCR purification kit [30], and each library eluted with 25 μl H_2_O.

Library concentrations were determined with Qubit dsDNA HS Assay kit [34], and library molecular size distributions determined using Bioanalyzer. The optimal amounts of libraries were then determined as per the Illumina qPCR quantification guide [11], and libraries sequenced at concentrations of 1.3 to 2.3 nM.

### Sequence processing and alignment

All alignments were made against the TAIR10 version of the *Arabidopsis* genome [12]. Prior to alignment, all reads within a library had the TS oligo sequence removed. For experiment 1, only reads starting with “GGG” were accepted, and the “GGG” sequence was removed before alignment. For libraries that were barcoded (experiments 2 and 3) and often had mismatches in the barcode portion of the read, a custom Perl script was used to assign a barcode to a library only if 1) the barcode had the fewest mismatches compared to any other barcode, and 2) no more than three mismatches total were found [37]. Once a barcode was identified, both the adapter with the barcode, and the following “GGG” portion of the adapter was removed before alignment. For experiment 1, which sequenced 101 nucleotides as opposed to 51 nucleotides in experiments 2 and 3, the last 41 nucleotides were trimmed prior to alignment in order to make sequence lengths more comparable across samples. rRNA reads were removed, and only uniquely mapped reads were used for analyses. CapFilter, described below, was used to pre-process all sequence files prior to alignment, but after adapter removal. After applying CapFilter, all reads were aligned to the TAIR10 reference genome, using Tophat version 2.0.12 [38], with the parameter settings ‘--bowtie1 -N 2 -i 50 -I 5000’ and the ‘--segment-length’ option set to half the length of the aligned read for those libraries where reads were < 51 nucleotides.

### Strand invasion filtering

Strand invasion artifacts were removed post-alignment using the same procedure outlined in [9] with some minor modifications. Briefly, the nine nucleotides directly upstream of the mapped reads were pulled from the reference genome and this sequence was compared to the last nine nucleotides of the TS oligo used for preparing the library. The last three nucleotides of the reference genome sequence had to have no less than two of the three “G”s present in the TS oligo tail, and no more than two mismatches overall. This same alignment requirement was then applied to subsequences one to three nucleotides upstream and downstream of the read alignment start position, and if at least one subsequence matched the last 9 nucleotides of the TS oligo, then the read was considered as showing evidence of strand invasion.

### Quartile plotting

All annotated protein coding genes were partitioned into four evenly spaced quartiles, relative to the orientation of the gene, starting at 100 bp upstream of the TAIR10 TSS and ending at the TAIR10 annotated end of the gene [12]. Quartiles were ordered from the most 5′ (quartile 1) to the most 3′ (quartile 4). The total number of reads whose 5′-most base resided within a given quartile was then calculated, and the percent of genes with the majority of reads found within a quartile was plotted.

### Peak identification and G’ filtering with CapFilter

CapFilter is a two-step process used to identify high confidence peaks. The first step pre-processes reads prior to alignment by trimming the first nucleotide of a given sequence and modifying the FASTQ identifier to keep track of this nucleotide. This FASTQ file is then aligned against the reference, and the subsequent BAM file produced by TopHat [38]. This BAM file is then filtered for strand invasion artifacts, and peak identification is performed using a previously developed peak calling program [4]. CapFilter then takes as input the BAM file, the generated peak file, and the reference genome sequence, and generates as output a final peak file containing high confidence TSS peaks. For this, CapFilter enumerates the percent of reads within a peak that had a 5′ unencoded “G” removed (based on the nucleotide identified in the “qname” field of the SAM alignment). CapFilter then creates a final peak file with high confidence peaks and adds an additional “%-Capped” column to the output to denote the percent of reads which had a 5′ unencoded “G”. Each unencoded “G” was identified by comparing the first base of the FASTQ file (which was removed prior to alignment) to the reference genome nucleotide that was one base pair upstream of the mapped read.

### Read correlation analysis

The total number of reads assigned to each peak was calculated using the same procedure outlined above for G’-filtering, and these totals were normalized to the number of reads per million (RPM) found within a given library. For all pairwise comparisons, all peaks were then paired based on overlapping coordinates; only those peaks that could be paired uniquely between libraries were compared. A Spearman’s rank correlation analysis was then performed for all peaks that could be paired between each library.

### Peak mode analysis

For all pairwise comparisons, all peaks were paired based on overlapping start/end coordinates. Only those peaks which could be paired uniquely were used, and the median and average distance between the identified peak modes was calculated. For those peaks in nanoCAGE-XL that were comparable to PEAT peaks (overlapping), only peaks within 500 bases of the TAIR10 annotated TSS or within the 5′ UTR were used for this comparison based on the criteria for PEAT peak selection [3]. For all comparisons within nanoCAGE-XL data uniquely paired peaks from all regions were used prior to filtering. After filtering, only uniquely paired peaks that had at least 50 % of reads beginning with an unencoded “G” were considered.

### Availability of supporting data

A detailed experimental protocol for nanoCAGE-XL is provided in Additional file 1. All raw sequencing data and TSS peak calls (made for each library individually and for each experiment combined) supporting the results of this article are available at Short Read Archive in [http://www.ncbi.nlm.nih.gov/sra] with accession [PRJNA270670]. The Perl script for assigning barcodes is publicly available as an open source command-line tool at [37]. The CapFilter program for identifying high confidence peak calls is publicly available as an open source command-line tool [37].

## Additional files

Additional file 1:
**Step by step description of the entire nanoCAGE-XL protocol.**
Additional file 2: Figure S1. Effect of rRNA depletion on nanoCAGE librariy profile. Panel a: Library constructed with total RNA as template. Panel b: Library constructed with Ribo-Zero depleted RNA as template. **Figure S2:** Examples of nanoCAGE TSS peak distribution before and after G’-filtering for experiments 2 and 3. **Figure S3:** Examples of CAGE TSS peak distributions before and after G’-filtering. Additional file 3: Table S1. Percent of reads with mismatch at the 1^st^ base (% X1) and 2^nd^ base (% *X*2). Additional file 4: Table S2. Summary of Peak Locations when applying G’ Filtering at a 50 % Cutoff. Additional file 5: Table S3. Spearman’s Rho for pairwise comparisons of peak RPMs.
