# Supplementary material for: NanoCAGE-XL and CapFilter: an approach to genome wide identification of high confidence transcription start sites
Source: BMC Genomics. 2015 Aug 13;16(1):597. doi: 10.1186/s12864-015-1670-6 (PMC4534009; doi:10.1186/s12864-015-1670-6)
Supplement: Additional file 1: — Step by step description of the entire nanoCAGE-XL protocol. [file 12864_2015_1670_MOESM1_ESM.docx]

**nanoCAGE-XL Protocol**

All reagents were from Sigma-Aldrich (<https://www.sigmaaldrich.com>) and mixtures were prepared on ice using nuclease-free plastics and reagents, unless otherwise noted.

**Primer Sequences Used**

Experiment 1

Template switching (TS): 5’-TAG TCG AAC TGA AGG TCT CCA GCA rGrGrG-3’

Reverse transcription (RT): 5’-TAG TCG AAC TGA AGG TCT CCG AAC CGC TCT TCC GAT CTN NNN NN-3’

Forward second-strand: 5’-TAG TCG AAC TGA AGG TCT CCA GC-3’

Reverse second-strand: 5’-TGA CGT CGT CTA GTC GAA CTG AAG GTC TCC GAA CC-3’

Forward library PCR: 5’-AAT GAT ACG GCG ACC ACC GAG ATC TAC ACT AGT CGA ACT GAA GG-3’

Reverse library PCR: 5’-CAA GCA GAA GAC GGC ATA CGA GAT CGG TCT CGG CAT TCC TGC TGA ACC GCT CTT CCG ATC T-3’

Sequencing primer: 5’-TAG TCG AAC TGA AGG TCT CCA GCA-3’

Experiment 2

Template switching-1 (TS-1): 5’-TAG TCG AAC TGA AGG TCT CCA GCA ATC GTG GCT ATA rGrGrG-3’

Template switching-2 (TS-2): 5’-TAG TCG AAC TGA AGG TCT CCA GCA GAT CGA GCT ATA rGrGrG-3’

Template switching-3 (TS): 5’-TAG TCG AAC TGA AGG TCT CCA GCA TCG AGC GCT ATA rGrGrG-3’

Reverse transcription (RT): same as Exp.1

Forward second-strand: same as Exp. 1

Reverse second-strand: same as Exp. 1

Forward library PCR: same as Exp. 1

Reverse library PCR: 5’-CAA GCA GAA GAC GGC ATA CGA GAT GTG ACT GGA GTT CAG ACG TGT GCT CTT CCG ATC T-3’

Sequencing primer: same as Exp. 1

Experiment-3

Template switching-1 (TS-1): 5’-TAG TCG AAC TGA AGG TCT CCA GCA CGA TGT rGrGrG-3’

Template switching-2 (TS-2): 5’-TAG TCG AAC TGA AGG TCT CCA GCA TGA CCA rGrGrG-3’

Template switching-3 (TS-3): 5’-TAG TCG AAC TGA AGG TCT CCA GCA GCC AAT rGrGrG-3’

Template switching-4 (TS-4): 5’-TAG TCG AAC TGA AGG TCT CCA GCA CAG ATC rGrGrG-3’

Template switching-5 (TS-5): 5’-TAG TCG AAC TGA AGG TCT CCA GCA CTT GTA rGrGrG-3’

Template switching-6 (TS-6): 5’-TAG TCG AAC TGA AGG TCT CCA GCA CCG TCC rGrGrG-3’

Reverse transcription (RT): same as Exp. 1, Exp. 2

Forward second-strand: same as Exp. 1, Exp. 2

Reverse second-strand: same as Exp. 1, Exp. 2

Forward library PCR: same as Exp. 1, Exp. 2

Reverse library PCR: same as Exp. 2

Sequencing primer: same as Exp. 1, Exp. 2

**First Strand cDNA synthesis**

Prepare Mix-1 by combining:

4 µl of primer/sorbitol//trehalose solution prepared as in (Salimullah et al., 2011)

x µl (~200 ng) of rRNA-depleted RNA

x µl of H_2_O to a final volume of 8 µl.

Incubate at 65^o^C for 10 min in a thermocycler, and cooled in ice water bath for 5 min.

Prepare Mix-2 by combining:

8 µl of 5 X Prime Script buffer

2.5 µl of 10 mM dNTPs

4 µl of 0.1 M DTT

6 µl of 5 M Betaine

4 µl of Prime Script Enzyme

x µl of H_2_O to a final volume of 32 µl

Combine Mix-1 and Mix-2, and incubate 10 min at 22^o^C, 30 min at 40^o^C, and 15 min at 75^o^C; snap cool in ice water bath for 5 min. Purify Agencourt RNA Clean XP magnetic beads (Beckman Coulter; <https://www.beckmancoulter.com>) following the manufacturer’s instructions. Elute with 80 µl H_2_O.

**Quantitative Real-Time PCR**

Using SYBR Premix Ex Taq kit (TaKaRa; <http://www.clontech.com/takara>), for each sample, prepare in triplicate 8.5 µl mix by combining:

5.0 µl of 2X *SYBER* Premix *Ex* *Taq*

0.1 µl of 10 µM forward second-strand PCR primer

0.1 µl of 10 µM reverse second-strand PCR primer

0.2 µl of 50X ROX Reference Dye

3.1 µl of H2O

Add 1.5 µl of purified cDNA or water (as negative control). Run PCR for 1 cycle at 95^o^C for 1 min; 30 cycles of 95^o^C for 15 sec, 65^o^C for 10 sec, 68^o^C for 2 min; hold at 12^o^C. Determine the cycle threshold for each sample (usually 13-16 cycles) and negative control (usually no amplification), and calculate the optimal number of cycles by adding 4 (usually 17-20 cycles).

**Second-Strand cDNA Synthesis**

Prepare 400 µl of PCR mix by combining:

40 µl of X10 *Ex* *Taq* buffer

8 µl of 10 mM dNTPs

4 µl of 10 µM forward second-strand PCR primer

4 µl of 10 µM reverse second-strand PCR primer

2 µl of *Ex* *Taq* polymerase

282 µl of H_2_O

60 µl of cDNA

Amplify as in real-time PCR using the determined number of cycles. Purify with Agencourt AMPure XP beads (Beckman Coulter; <https://www.beckmancoulter.com>) following the manufacturer’s instructions. Elute with 120 µl of H_2_O. Determine concentration by NanoDrop spectrophotometer and by Qubit dsDNA HS Assay Kit (Molecular Probes; <http://www.lifetechnologies.com>) following the manufacturer’s instructions. Concentration should be ≥10 ng per µl by Qubit.

Dilute samples to 10 ng per µl by the Qubit values

Note: High rRNA content may result in the generation of ‘spiky’ libraries and a loss of sequencing information (see Supplementary Figure 1). To verify the library profile, second-strand cDNA can be prepared in a 10% format (40 µl) and analyzed by Agilent Bioanalyzer before performing a full-scale format.

**Addition of Sequencing Adapters**

For each library, prepare 700 µl of PCR mix:

140 µl of HF buffer

14 µl of 10 mM dNTPs

14 µl of forward library primer

14 µl of reverse library primer

7 µl of Phusion polymerase (Thermo Scientific; http://www.thermoscientificbio.com)

399 µl of H_2_O

112 µl of diluted second-strand cDNA

Run PCR for 1 cycle at 98^o^C for 1 min; 1 cycle at 98 ^o^C for 15 sec, 55 ^o^C for 10 sec, 68 ^o^C for 2 min; 8 cycles of 98^o^C for 15 sec, 65^o^C for 10 sec, 68^o^C for 2 min; hold at 12^o^C.

**Exo I Nuclease Digestion**

Combine samples, and add 4 µl of Exo I, mix, incubate at 37^o^C for 30 min.

To purify the resulting product, mix with 5 volumes (3.5 ml) of PB buffer (Qiagen; <http://www.qiagen.com>) and purify through a single column using a PCR-purification kit (Qiagen; <http://www.qiagen.com>) following the manufacturer’s instructions. Elute with 25 µl of water.

**Library quantification**

Determine concentration by NanoDrop spectrophotometer (usually ~50-100 ng per µl). Dilute 1 µl to 15 µl with water, and analyze library size by Agilent Bioanalyzer (usually ~1,400-1,700 bp). Libraries are used for qPCR analysis without further dilution, and library molarity calculated based on the obtained values (usually ~2-4 nM). Libraries were sequenced at concentrations of 1.3 to 2.3 nM.
