# Supplementary material for: NanoCAGE-XL and CapFilter: an approach to genome wide identification of high confidence transcription start sites
Source: BMC Genomics. 2015 Aug 13;16(1):597. doi: 10.1186/s12864-015-1670-6 (PMC4534009; doi:10.1186/s12864-015-1670-6)
Supplement: Additional file 2: Figure S1. — Effect of rRNA depletion on nanoCAGE librariy profile. Panel a: Library constructed with total RNA as template. Panel b: Library constructed with Ribo-Zero depleted RNA as template. Figure S2: Examples of nanoCAGE TSS peak distribution before and after G’-filtering for experiments 2 and 3. Figure S3: Examples of CAGE TSS peak distributions before and after G’-filtering. [file 12864_2015_1670_MOESM2_ESM.docx]

**
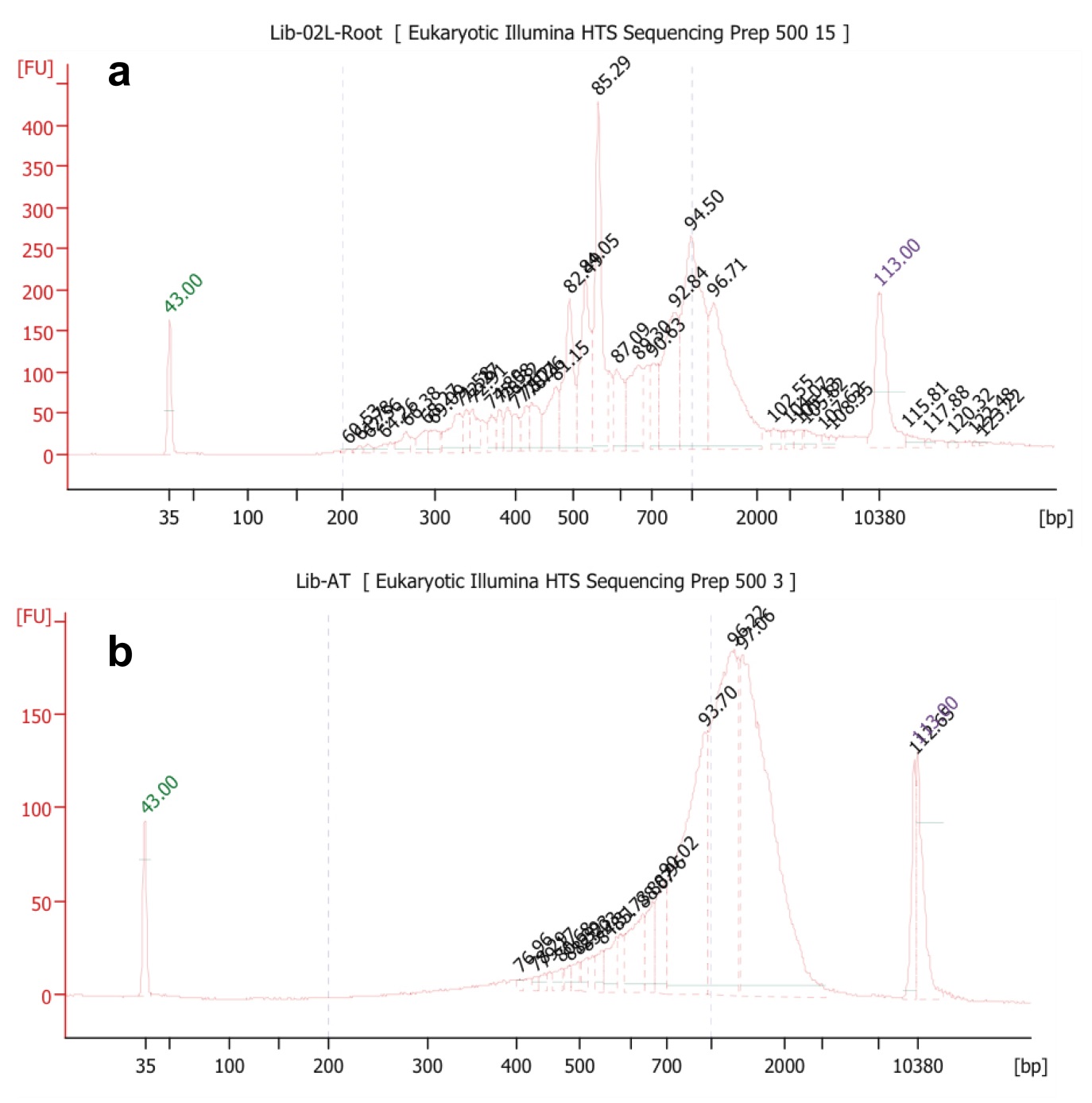
**

**Figure S1. Effect of rRNA depletion on nanoCAGE-XL library profile**

**Panel a: Library constructed with total RNA as template**

**Panel b: Library constructed with Ribo-Zero depleted RNA as template.**

**
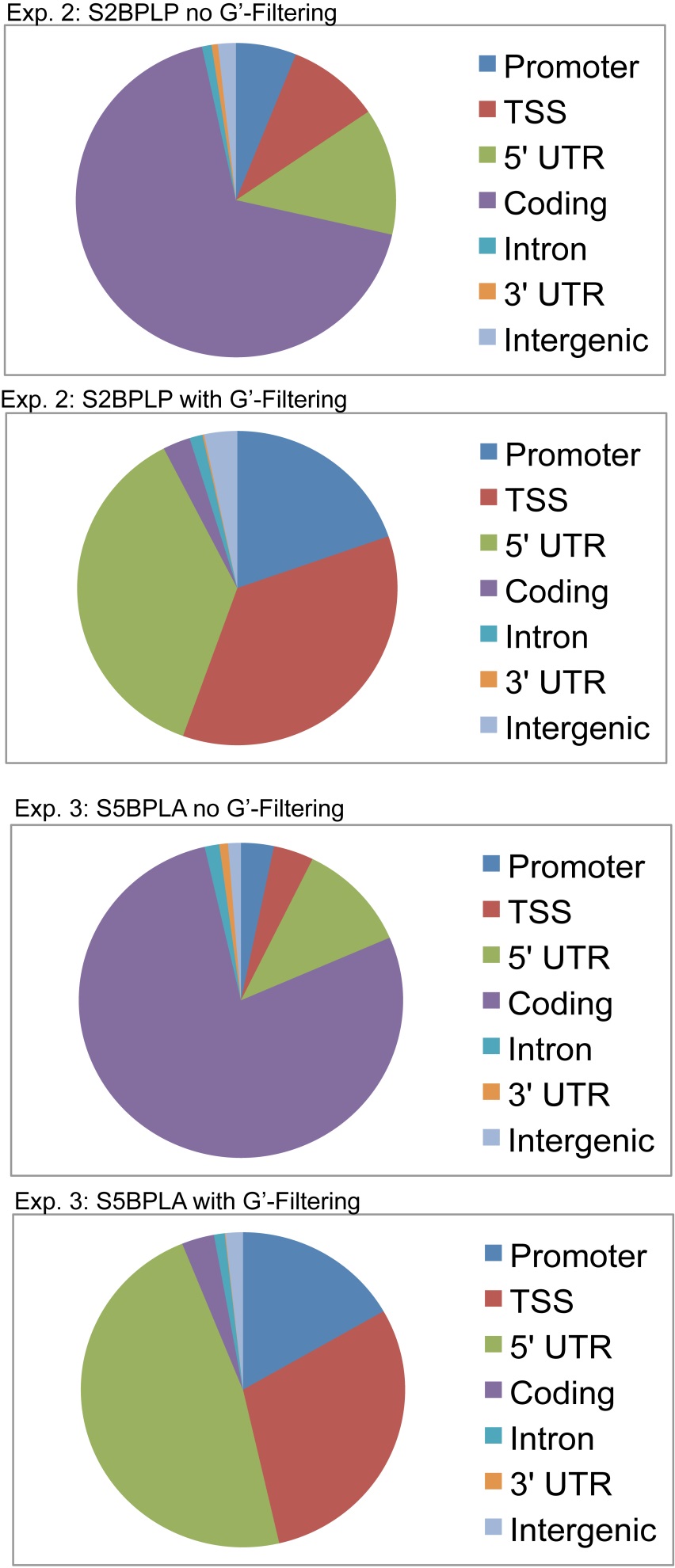
**

**Figure S2.** **Examples of nanoCAGE TSS peak distribution before and after G’-filtering for experiments 2 and 3.**

**
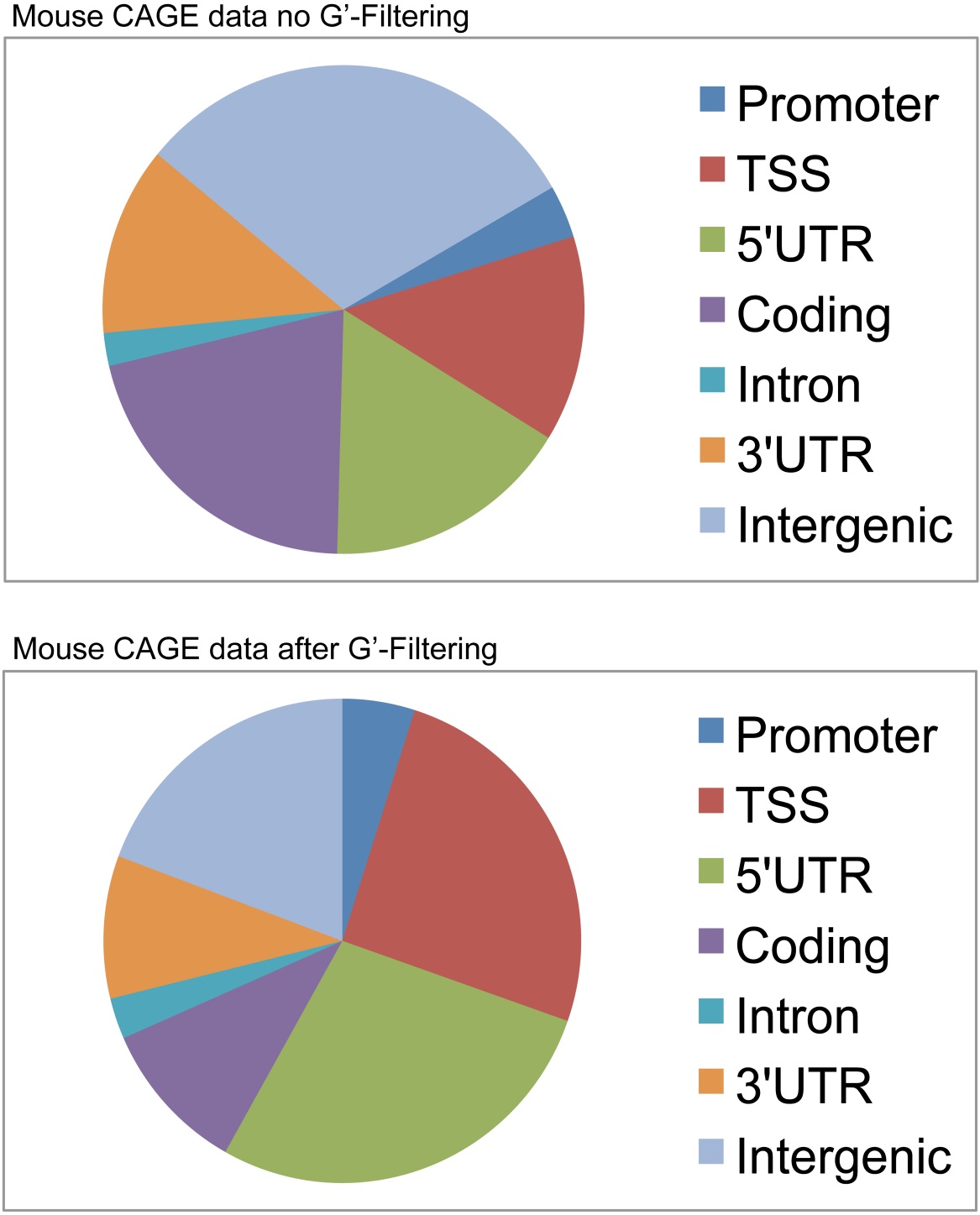
**

**Figure S3. Examples of CAGE TSS peak distributions before and after G’-filtering**
